# Biomimetic model for computing missing data imputation and inconsistency reduction in pairwise comparisons matrices

**DOI:** 10.1371/journal.pone.0329171

**Published:** 2025-08-07

**Authors:** Waldemar W. Koczkodaj, Witold Pedrycz, Alexander Pigazzini, Laura P. Pigazzini

**Affiliations:** 1 Department of Computer Science, Laurentian University, Sudbury, Ontario, Canada; 2 Department of Electrical and Computer Engineering, University of Alberta, Edmonton, Alberta, Canada; 3 Department of Mathematics, Mathematical and Physical Science Foundation, Slagelse, Denmark; 4 Liceo Statale A. Banfi, Liceo Scientifico Tradizionale, Vimercate, Italy; Amity University, INDIA

## Abstract

A biomimetic model is presented to compute missing data imputation and reduce inconsistencies in pairwise comparisons matrices. The proposed regeneration method emulates three primary phases of a biological process: identifying the most damaged areas (by identifying inconsistencies in the pairwise comparison matrix), cell proliferation (filling in missing data), and stabilization (optimization of global consistency). An iterative algorithm is employed to correct inconsistencies and compute missing data imputations within the pairwise comparison matrix. The results demonstrate that the biomimetic approach is robust and reliably converges to a consistent solution.

## 1 Introduction

Nature has inspired numerous researchers to develop materials, structures, tools, mechanisms, processes, algorithms, and methods.

Self-healing approaches are presented in [1]. A review of inspiration by nature for the potential development of biomimicry appeared in [2]. The pairwise comparison method (PC or PCs, depending on the context) plays a significant role in assessments, subjective measurements, and decision-making. In various applied sciences, such as engineering, physics, and medicine, physical measurements-like length, weight, area, volume, and temperature are of fundamental importance. However, many researchers who rely on these physical measurements may not realize how frequently subjective measurements are utilized in practice. In fact, subjective measures can often be applied to a broader range of entities than objective measures. For example, evaluating student performance is necessary to award academic degrees, which are crucial to our civilization. Most academic exams rely on rating scales, and pairwise comparisons can enhance these scales used to assess the learning process. The most important challenges to address for the pairwise comparison method include the following:

1. reduction of inconsistency (not necessarily to zero),
2. finding the nearest consistent PC matrix by optimization,
3. computing the imputation of missing data, known as the regeneration problem.

**Fig 1:**
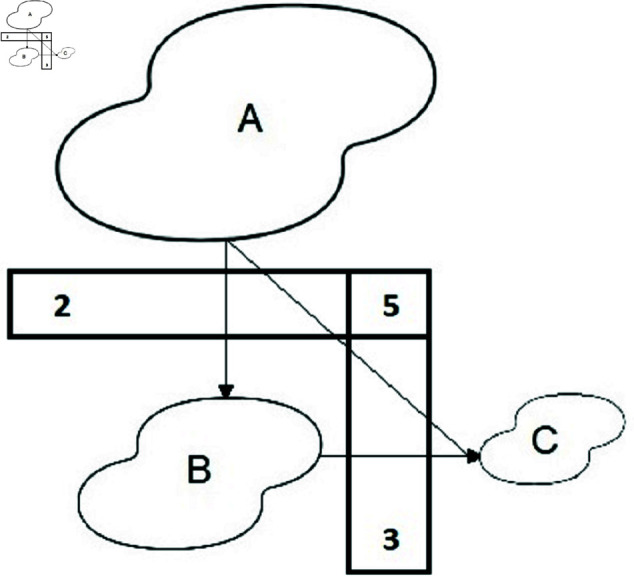
Inconsistency concept for a triad (2, 5, 3).

The importance of reducing inconsistency in pairwise comparisons (PC) matrices has led to a search for methods to measure and locate it. The inconsistency concept is illustrated by Fig 1.

Traditionally, values of pairwise comparisons are stored in square PC matrices (see [3]). PC matrices have all strictly positive real elements. Each element of a matrix represents a ratio between two entities. Entities may be physical objects (e.g., volumes of river rocks) or abstract concepts (software attributes). More often than not, PCs are used for decision-making. The quality of decisions can be compromised by two main issues: *inconsistency* in the evaluations and *missing PC matrix elements*:

- *inconsistency* in a triad (*i*,*j*,*k*) is quantified by the index
  $$K_{ijk}=\min\left(\left|1-\frac{a_{ik}}{a_{ij}\cdot a_{jk}}\right|,\left|1-\frac{a_{ij}\cdot a_{jk}}{a_{ik}}\right|\right),$$
  which measures the deviation from the consistency condition $a_{ik}=a_{ij}\cdot a_{jk}$. Its value is non-zero, when appropriate assessments within a PC matrix are intransitive. For example, if *a*_*ij*_ = 2 and *a*_*jk*_ = 3, then the element *a*_*ik*_ should be $a_{ij}\cdot a_{jk}=6$. If *a*_*ik*_ differs from this value, inconsistency arises.
- *missing data imputation* when some elements of the PC matrix are unknown.

The pairwise comparisons method tolerates or even utilizes inconsistency in assessments. By analyzing inconsistency, we may improve data acquisition or replace the least probable value in a triad by assuming that it is missing and using the proposed missing data imputation approach.

Inconsistency is related to the number of comparisons. For a PC matrix *M* of the size *n*, it is n·(n−1)/2 in the upper triangle while the minimum number of comparisons is *n*–1 (for details, see [4]). When there is more data than the required minimum, inconsistency may occur. This principle was addressed in [4] and it is illustrated here in Fig 1. Values 2, 5, and 3 are labels for the arrows between entities. It shows our (subjective) assessment of the entity A area when compared with B as 2. Value 3 shows that the area of B is three times bigger than C. If the ratios between A and B and C were accurate, the ratio of areas A and C should be 2·3=6. However, our assessment is 5 and illustrates inconsistency. It is necessary to note the use of the conditional phrase "If the ratios between A and B and C were accurate" in the above text. We do not assume that any of the ratios A/B, B/C or even A/C are accurate. This is why ratio estimations can be inconsistent, and we need to compute the inconsistency.

Even when assessments are fully consistent, there is no guarantee that the resulting priority vector will be accurate. For example, a PC matrix filled entirely with 1s is completely consistent, but it does not provide useful prioritization information. To address this, recent research has introduced the concept of entropy of the priority vector to measure the information content of inconsistent PC matrices (see [5]). This approach demonstrates that the entropy of priority vectors for consistent matrices follows a normal distribution.

According to [6]:

biomimetic, also known as biomimicry, is defined as the imitation of biological processes or models from nature aiming to solve various complex biological problems, such as drug production in biomedical applications and the characterization of nanostructurated biohybrid materials.

In practical terms, the use of pairs in biology is substantial. It is not only applicable to sex but also to legs, hands, eyes, ears, and more. For this reason, we may consider the pairwise comparisons method as a crown example of biomimicry. The pairwise comparisons method has much to learn from biomimetics. This article serves as a call to action. Using biomimetics in pairwise comparisons method is highly advisable.

We propose a model inspired by the process of biological tissue regeneration, in which an organism repairs its damaged tissues. Tissue engineering (see, [7–18]) aims to regenerate or replace damaged tissues with new cells. A crucial component of this process is the scaffold. It is a three-dimensional structure that provides support for cells by mimicking the extracellular matrix (ECM) (see [19]). The biomimetic approach in scaffold design enhances cellular integration, often inspired by biological mechanisms (see [20]) designed to achieve controlled interactions, as seen in nanoparticle uptake models (see [13]). These scaffolds and biomimetic devices address challenges in tissue repair and inflammation control, as explored in the selective cytopheretic device for sepsis (see [12]). The application of these design principles emphasizes collaborative efforts aimed at optimizing tissue regeneration and scaffold-based therapies.

Several studies have addressed inconsistency reduction and missing data imputation in PC matrices. Numerous methods (e.g., scaling techniques [21], inconsistency minimization algorithms [22], or traditional statistical approaches [23]) focus on correcting inconsistencies but do not simultaneously handle missing data imputation. Some recent approaches, such as those based on non-linear optimization [24] and weighted least squares models [25], estimate missing data imputation by minimizing inconsistency. However, they do not integrate biological principles for a dynamic interaction between the two problems. Furthermore, classifications such as in [23] list categories of methods for PC matrices (e.g. scaling methods, statistical methods) but do not include biomimetic approaches.

Our approach differs from other approaches. It is inspired by the process of tissue regeneration, which allows us to address inconsistencies and missing data imputation simultaneously and iteratively, ensuring convergence towards consistent solutions.

Recent advances in missing data imputation have explored diverse domains and methodologies. In the clinical setting, the DACMI Challenge [26] has demonstrated the effectiveness of machine learning approaches such as:

- gradient boosting, LightGBM - (https://github.com/microsoft/LightGBM), documentation available on https://lightgbm.readthedocs.io/en/stable/.
- statistical techniques (e.g., MICE, see [27], available as an *R* package at https://cran.r-project.org/web/packages/mice/index.html).

They handle incomplete temporal data.

Missing data imputation methodologies, such as multiple imputation, MI, see as reference [28], and maximum likelihood estimation, have been applied in numerous disciplines to fill numerical and categorical gaps [29]. However, these approaches prioritize statistical plausibility and scalability and do not address the specific challenges of PC matrices, where logical consistency is critical despite missing or conflicting entries.

The proposed biomimetic regeneration model fills this gap by using iterative inconsistency correction, inspired by biological repair mechanisms, and through dimensionality reduction approaches (such as PCA, see *Appendix B*), ensures consistency in PC matrices entries, a requirement not satisfied by existing methods [30].

## 2 Tissue regeneration model for PC matrices

According to [31], *regeneration*, in biology, is the process by which some organisms replace or restore lost or amputated body parts.

### Biological concepts of regeneration

Tissue regeneration involves the following three main phases:

1. *identification of the damaged area*: the body identifies the damaged or missing areas of the tissue,
2. *cell proliferation*: cells proliferate to replace damaged cells, following a controlled growth process,
3. *remodelling and stabilization* of the new tissue integrates with the existing one, stabilizing and restoring the original functionality.

### Mathematical transposition into an algorithm for PC matrices

Transposing the above-mentioned process into a mathematical context requires the following iterative algorithm.

**Algorithm: regeneration of a PC Matrix**

1. Identify damaged areas in the matrix (inconsistencies),
2. Proliferate new values for missing entries,
3. Reshape and stabilize the matrix reducing global inconsistency.

## Tissue regeneration algorithm for PC matrices

*Input:* Pairwise comparison matrix *A* of size n×n, with missing and/or inconsistent entries.

1. *Step 1 (Identification of damaged areas):* The inconsistent triads, representing the "damaged" regions that need correction, are identified.
2. Proliferate new values for missing entries,
3. Reshape and stabilize the matrix reducing global inconsistency.
4. *Step 2 (Cellular proliferation):*
  The missing data imputation *a*_*ij*_ are initialized with the geometric mean of the involved triads (e.g., $a_{ij}^{\left(0\right)}=\sqrt{a_{ik}\;\cdot \;a_{kj}}$). Subsequently, they are iteratively updated by the formula $a_{ij}^{\left(t+1\right)}=a_{ij}^{\left(t\right)}+\eta \;\cdot \;\left(\frac{1}{n-2}\sum_{k\neq i,j}\frac{a_{ik}\;\cdot \;a_{kj}}{a_{ij}}-a_{ij}\right)$ until the value tends to stabilize.
5. *Step 3 (Matrix Stabilization):*
  The gradient method is applied to minimize $\sum (K_{ijk}{)}^{2}$. The algorithm continues to iterate until the overall inconsistency drops below a predefined threshold or a maximum number of iterations is reached, ensuring convergence to a more consistent matrix. For *Kii*, the 1/3 threshold is proposed as reasonable for most applications. Similarly to *p*–*value* in statistics, it is an arbitrary value often found by experimental research.

*Output:* Pairwise comparisons matrix $A^{\prime }$, consistent and complete. The inconsistency in the PC matrix and the previous three steps are discussed in the following.

### Step 1: Identification of damaged areas

1. *Computing of inconsistency index *Kii**_*ijk*_: For each triad of elements (*i*,*j*,*k*) in matrix *A*, the inconsistency index *Kii*_*ijk*_ is computed as:
  $$Kii_{ijk}=\min\left(\left|1-\frac{a_{ik}}{a_{ij}\;\cdot \;a_{jk}}\right|,\left|1-\frac{a_{ij}\;\cdot \;a_{jk}}{a_{ik}}\right|\right),$$
  or the equivalent exponential Koczkodaj-Szwarc formula (see, [32]):
  $$Kii(x,y,z)=1-e^{-|ln(\frac{y}{xz})|}$$
  This index quantifies the deviation from the ideal consistency condition $a_{ik}=a_{ij}\;\cdot \;a_{jk}$.
2. *Identification of inconsistent triads:* Identify the triads (*i* ,*j* ,*k* ) with the maximum value of *Kii*_*ijk*_, defining these areas as the most inconsistent in the matrix. These triads correspond to the "damaged zones" to be corrected.

### Step 2: Cell proliferation

1. *Initialization of missing entries:* For each missing value *a*_*ij*_, initialize the value $a_{ij}^{\left(0\right)}$ using an estimate based on existing values. A possible estimate is the geometric mean of the products of known pairs:
  $$a_{ij}^{\left(0\right)}=\sqrt{a_{ik}\;\cdot \;a_{kj}}\;\text{for every }k\neq i,j\;\text{and}\;i\neq j,\;\text{with}\;i,j,k\in 1,2,..,n.$$
  This is done for each triad with a missing value. Finally, a geometric mean of the values obtained is computed and inserted in place of the missing value. This is analogous to the cell proliferation that fills the void created by the injury.
2. *Iterative update:* for each iteration *t*, update the values of missing entries and inconsistent triads to reduce inconsistency:
  $$a_{ij}^{\left(t+1\right)}=a_{ij}^{\left(t\right)}+\eta \;\cdot \;\left(\frac{1}{3}\sum_{k\neq i,j}\left(\frac{a_{ik}^{\left(t\right)}\;\cdot \;a_{kj}^{\left(t\right)}}{a_{ij}^{\left(t\right)}}\right)-a_{ij}^{\left(t\right)}\right)$$
  where η is a learning parameter controlling the update speed. This step is similar to the controlled growth of new tissue to repair damage.

### Step 3: Iteration and stabilization

1. *Consistency optimization:* The next step is to optimize the PC matrix to minimize global inconsistency. An optimization algorithm, such as the gradient method, is used to minimize the sum of the squares of the inconsistency indices:
  $$\text{minimize}\;\sum_{i,j,k}{\left(Kii_{ijk}^{\left(t\right)}\right)}^{2}$$
  Calculating the gradient with respect to each element *a*_*ij*_:
  $$\frac{\partial Kii_{ijk}}{\partial a_{ij}}=\frac{\partial }{\partial a_{ij}}\min\left(\left|1-\frac{a_{ik}}{a_{ij}\;\cdot \;a_{jk}}\right|,\left|1-\frac{a_{ij}\;\cdot \;a_{jk}}{a_{ik}}\right|\right)$$
  It updates the values of *a*_*ij*_ accordingly.
2. *Stabilization:* The algorithm continues iterating until the overall inconsistency $\sum_{i,j,k}Kii_{ijk}$ falls below a predefined threshold $\epsilon_{\text{min}}$ or a maximum number of iterations is reached. This step corresponds to the regenerated tissue, integrating it with the pre-existing tissue.

## 3 Analysis of the convergence and robustness of the algorithm

Consider a matrix $A\in {\mathbb{R}}^{n\times n}$, whose elements *a*_*ij*_ represent pairwise comparisons between entities *i* and *j*. The goal of the algorithm is to correct the inconsistency in the matrix *A*. We define the local inconsistency of a triad (*i*,*j*,*k*) by the index *K*_*ijk*_, as:


$$K_{ijk}=\min\left(\left|1-\frac{a_{ik}}{a_{ij}a_{jk}}\right|,\left|1-\frac{a_{ij}a_{jk}}{a_{ik}}\right|\right)$$

The global inconsistency *I*(*A*) is then the sum of the local inconsistencies for all triads in the matrix:


$$I(A)=\sum_{i,j,k}K_{ijk}$$

We reduce *I*(*A*) for each iteration until the inconsistency is acceptable, i.e., $I(A)\leq \varepsilon_{\min}$, where $\varepsilon_{\min}$ is a stopping threshold.

The elements *a*_*ij*_ of the PC matrix are iteratively updated according to this rule:


$$a_{ij}^{\left(t+1\right)}=a_{ij}^{\left(t\right)}+\eta \;\cdot \;\Delta a_{ij}^{\left(t\right)}$$

where $\Delta a_{ij}^{\left(t\right)}$ represents a correction based on the local inconsistency and η is a positive learning rate that controls the magnitude of the update. The term $\Delta a_{ij}^{\left(t\right)}$ can be expressed as:


$$\Delta a_{ij}^{\left(t\right)}=\frac{1}{3}\sum_{k\neq i,j}\left(\frac{a_{ik}^{\left(t\right)}a_{kj}^{\left(t\right)}}{a_{ij}^{\left(t\right)}}-a_{ij}^{\left(t\right)}\right)$$

The goal of this update rule is to correct the elements of the PC matrix that contribute the most to inconsistency, iteratively reducing *I*(*A*).

To demonstrate that the algorithm converges, we need to show that the function *I*(*A*) is non-increasing, meaning that at each step the global inconsistency decreases:


$$I(A^{\left(t+1\right)})\leq I(A^{\left(t\right)})$$

In order to reduce *I*(*A*) at each iteration, the update of the matrix elements follows the gradient descent method. The gradient of *I*(*A*) with respect to *a*_*ij*_ is given by:


$$\nabla_{a_{ij}}I(A)=\frac{\partial I(A)}{\partial a_{ij}}$$

The update of *a*_*ij*_ is done in the direction opposite to the gradient:


$$\Delta a_{ij}^{\left(t\right)}=-\nabla_{a_{ij}}I(A^{\left(t\right)})$$

This ensures that the global inconsistency *I*(*A*) decreases as the update rule modifies *a*_*ij*_ in the direction that reduces *I*(*A*) as quickly as possible.

In order for the algorithm to converge, the variation $\eta \;\cdot \;\Delta a_{ij}$ cannot be too large. If η is too large, we might overshoot the minimum of the function *I*(*A*) in a single step, causing instability or divergence.

Let us consider a second-order approximation for *I*(*A*) in terms of *a*_*ij*_:


$$I(A^{\left(t+1\right)})\approx I(A^{\left(t\right)})+\nabla_{a_{ij}}I(A^{\left(t\right)})\;\cdot \;(a_{ij}^{\left(t+1\right)}-a_{ij}^{\left(t\right)})+\frac{1}{2}\nabla_{a_{ij}}^{2}I(A)\;\cdot \;(a_{ij}^{\left(t+1\right)}-a_{ij}^{\left(t\right)}{)}^{2}$$

Substituting the update rule $a_{ij}^{\left(t+1\right)}=a_{ij}^{\left(t\right)}-\eta \nabla_{a_{ij}}I(A^{\left(t\right)})$, we get:


$$I(A^{\left(t+1\right)})\approx I(A^{\left(t\right)})-\eta {\left(\nabla_{a_{ij}}I(A^{\left(t\right)})\right)}^{2}+\frac{1}{2}\eta^{2}\nabla_{a_{ij}}^{2}I(A)\;\cdot \;{\left(\nabla_{a_{ij}}I(A^{\left(t\right)})\right)}^{2}$$

In order for $I(A^{\left(t+1\right)})\leq I(A^{\left(t\right)})$, the second term must be negative and dominate the third term. This leads us to the condition:


$$\eta \leq \frac{2}{\max_{i,j}\nabla_{a_{ij}}^{2}I(A)}$$

where $\nabla^{2}I(A)$ represents the second derivative of the function, specifically indicating how quickly the gradient of *I*(*A*) changes as a function of the matrix elements *a*_*ij*_. The second derivative provides insight into the local behaviour of the function and helps determine how to update the matrix to reduce inconsistency. The second derivative of the function *I*(*A*) with respect to *a*_*ij*_ is:


$$\nabla_{a_{ij}}^{2}I(A)=\frac{\partial^{2}I(A)}{\partial a_{ij}^{2}}$$

This expression represents how the rate of change (the gradient) of the global inconsistency function *I*(*A*) itself changes as we adjust *a*_*ij*_. In simpler terms, it tells us how sharply or smoothly the function changes in response to variations in the matrix element *a*_*ij*_.

Interpretation of the Second Derivative:

– If $\nabla_{a_{ij}}^{2}I(A)>0$, the function is locally increasing (convex behaviour), and a small change in *a*_*ij*_ will cause the function to increase in that direction.
  – If $\nabla_{a_{ij}}^{2}I(A)<0$, the function is locally decreasing (concave behaviour), and a change in *a*_*ij*_ will cause the function to decrease.

The condition $\eta \leq \frac{2}{\max_{i,j}\nabla_{a_{ij}}^{2}I(A)}$ ensures that the update step is approximately sized to avoid overshooting the minimum or causing oscillations. This guarantees that each step of the algorithm moves in a controlled way toward minimizing the inconsistency function *I*(*A*).

Since η is chosen to satisfy the above condition, the term $-\eta \left(\nabla_{a_{ij}}I(A^{\left(t\right)}\right){)}^{2}$ dominates the second-order term. Therefore:


$$I(A^{\left(t+1\right)})\leq I(A^{\left(t\right)})$$

which proves that the global inconsistency decreases or remains constant at each step.

The algorithm is robust to small perturbations in the initial values and parameters. Small variations in the initial values $a_{ij}^{\left(0\right)}$ lead to only small variations in the final behaviour of the algorithm, as successive corrections gradually reduce the inconsistency until a stable solution is reached.

The proposed algorithm converges under the conditions established for η, ensuring a continuous reduction of the global inconsistency *I*(*A*) at each iteration. The parameter η must be chosen based on the maximum curvature of the inconsistency function *I*(*A*) to ensure stability and avoid oscillations.

The convergence properties of the algorithm are not only mathematically proven but also have direct implications for real-world applications. In decision-making systems where consistency is critical (e.g., medical diagnostics or engineering safety assessments), the guaranteed convergence ensures that the algorithm does not indefinitely oscillate between inconsistent states. For instance, in a robotic control system requiring real-time adjustments of priorities (as in [33]), the bounded global inconsistency *I*(*A*) guarantees stable operational parameters even in dynamic environments.

The learning rate η plays a dual role in balancing convergence speed and numerical stability:

- Low η (e.g., η=0.05): Ensures smooth, stable updates but may require more iterations, suitable for scenarios where precision is prioritized over speed (e.g., clinical decision-making).
- High η (e.g., η=0.2): Accelerates convergence but risks overshooting optimal values, acceptable in non-critical applications (e.g., inventory prioritization) where computational efficiency is paramount.

In applications, η must be calibrated based on the problem’s sensitivity to noise. For example, in the medical example cited in [12], where tissue-regeneration-inspired matrices are used to prioritize treatments, a conservative η prevents destabilizing fluctuations in critical thresholds.

## The lower bound of inconsistency

The global inconsistency *I*(*A*) is a sum of non-negative terms. It is bounded below by zero:


$$I(A)=\sum_{i,j,k}K_{ijk}\geq 0$$

Therefore, there exists a lower bound $\epsilon_{\text{min}}$ (fixed at will), such that *I*(*A*) cannot fall below this threshold. If the algorithm continues to reduce inconsistency but cannot drop below $\epsilon_{\text{min}}$, it must stop once *I*(*A*) approaches this value.

## Convergence to a stable solution

To prove that the algorithm will converge to a stable solution, consider the sequence $\{I(A^{\left(t\right)}){\}}_{t\geq 0}$. We have already shown that this sequence is non-increasing and bounded below by $\epsilon_{\text{min}}$. By the convergence theorem of a bounded and monotonic sequence, {*I*(*A*^(*t*)^)} will converge to a limit value *I* .

The algorithm follows the gradient descent that minimizes *I*(*A*). We can conclude that the algorithm converges to a PC matrix *A*  with an inconsistency level *I* , which can be zero or an arbitrary acceptable value:


$$\lim_{t\to \infty }I(A^{\left(t\right)})=I^{*}$$

If the algorithm approaches *I*  by a small arbitrary value, we consider *A*  as a consistent or nearly consistent matrix, resolving the inconsistency reduction problem.

The “arbitrary level” for inconsistency is similar to the concept of “p-value” in statistics. We are aware that it is not an ideal solution, but it follows the “good enough is perfect” approach proposed by Herbert A. Simon. It is a part of his *bounded rationality principle* that was a major contribution to earning him both Turing and Nobel prizes.

Even if the initial estimates of the missing data imputation may not be perfect, the biomimetic approach is robust and tends to correct them by regeneration. The initial inaccuracy is improved through iteration and optimization. It mimics the tissue regeneration of how an organism corrects initial mistakes during tissue repair.

## Computational complexity

The computational complexity of the proposed method is $\text{O}({\text{n}}^{3})$, where *n* represents the dimension of the matrix (i.e., the number of elements being compared). It is determined by:

1. the algorithm computes inconsistencies for every triad in a PC matrix indexed by (*i*,*j*,*k*); the number of all possible triads in an n×n PC matrix is (n3), which is $\text{O}({\text{n}}^{3})$,
2. during each iteration, the values of the inconsistent elements in the matrix are updated to reduce the global inconsistency. The number of elements in a matrix is $\text{O}({\text{n}}^{2})$, and in each iteration, these values must be computed and updated.

Step 1 dominates the computational complexity, since each triad is evaluated in every iteration. However, no other mathematically correct method can avoid processing all triads since the number of all triads grows with ${\text{n}}^{3}$. Fortunately, the PC matrix size is small (not exceeding 8 in most methodologies based on PC method).

The number of iterations required for convergence depends on the initial level of inconsistency in the matrix and the threshold value $\epsilon_{\text{min}}$ chosen for acceptable inconsistency. A very small threshold requires more iterations, increasing.

For PC matrices, the elements below the main diagonal are the reciprocals of the elements above the diagonal. Consequently, we can compute and update only the upper half of the matrix, leading to savings in both computational time and memory usage. Furthermore, for large matrices, optimization techniques based on approximation methods, such as iterative methods (e.g., gradient descent with momentum), low-rank approximations (e.g., singular value decomposition or matrix factorization), can be applied to accelerate convergence, or principal component analysis (PCA) for dimensionality reduction.

While the proposed biomimetic algorithm exhibits $\text{O}({\text{n}}^{3})$ complexity due to triad evaluation, this aligns with other specialized methods for PC matrix inconsistency reduction and imputation. For example, nonlinear optimization approaches (e.g., [24]) and weighted least squares models (e.g., [25]) also require $\text{O}({\text{n}}^{3})$ operations due to matrix inversion or gradient-based steps. However, our iterative update rule avoids full matrix inversion, offering practical efficiency gains for small-to-medium-sized PC matrices (n≤8).

For large matrices (*n*>10), our method’s complexity can be reduced (via PCA, see *Appendix B*), comparable to low-rank approximation techniques. In contrast, general-purpose imputation methods like MICE [30] or LightGBM [26], while offering $\text{O}({\text{n}}^{2})$ asymptotic efficiency, fail to enforce PC matrix consistency constraints, a critical limitation for decision-making applications. Table 1 summarizes the theoretical and practical trade-offs between methods, highlighting the unique capability of our approach to simultaneously address inconsistency reduction and missing data imputation without sacrificing scalability through dimensionality reduction.

**Table 1:** Comparison of methods for inconsistency reduction and missing data imputation in pairwise comparison matrices.

Method	Complexity	Consistency	Handling
	O(·)	Guaranteed	Missing Data
Biomimetic (Proposed)	$\text{O}({\text{n}}^{3})$	Yes	Yes
Nonlinear Optim. [24]	$\text{O}({\text{n}}^{3})$	Yes	No (requires initialization)
Wtd. Least Squares [25]	$\text{O}({\text{n}}^{3})$	Yes	No
MICE/LightGBM [26,30]	$\text{O}({\text{n}}^{2})$	No	Yes

The presented biomimetic approach simultaneously computes inconsistencies and missing data imputation. We are unaware of any other method capable to do it this way.

## 4 A more comprehensive real-life example

There are two practical challenges associated with real-life examples of PC solutions.

1. Non-trivial problems, such as selecting a site for nuclear waste disposal, often take several years to resolve and can easily cost millions of dollars.
2. These problems are highly specialized, making them understandable only to a small group of domain experts.

For these reasons, we have used an example from [34]. It pertains to a crucial and urgent research topic: autism in children, which aligns with the scope of PLOS ONE.

### 4.1 Method

To avoid self-plagiarism (by copying the same example here), we use different values. However, it is just a matter of the labels of rows and columns of the PC matrix that make it a real-life example. Therefore, this research does not contain any studies on human participants or animals conducted by the authors.

### 4.2 Example

Let us optimize an incomplete PC matrix *A* of the size 5 by 5 by estimating the missing data imputation (based on the geometric means of the triads), and subsequently by applying the iterative algorithm to minimize the inconsistency.

Consider the following 5x5 PC matrix with two missing data imputation, *a*_14_ and *a*_25_:


$$A=[\begin{array}{ccccc} 1 & 3 & 5 & ? & \frac{1}{7}\\ \frac{1}{3} & 1 & 4 & 6 & ?\\ \frac{1}{5} & \frac{1}{4} & 1 & 2 & \frac{1}{9}\\ ? & \frac{1}{6} & \frac{1}{2} & 1 & \frac{1}{3}\\ 7 & ? & 9 & 3 & 1 \end{array}]$$

A missing value is in three triads with the missing value *a*_14_ and three triads with *a*_25_). The missing data imputation will be estimated using the geometric mean of each triad.

The estimation of *a*_14_ involved triads:

1. Triad (1, 2, 4): $\sqrt{a_{12}\;\cdot \;a_{24}}=\sqrt{3\;\cdot \;6}=\sqrt{18}\approx 4.24$
2. Triad (1, 3, 4): $\sqrt{a_{13}\;\cdot \;a_{34}}=\sqrt{5\;\cdot \;2}=\sqrt{10}\approx 3.16$
3. Triad (1, 5, 4): $\sqrt{a_{15}\;\cdot \;a_{54}}=\sqrt{\frac{1}{7}\;\cdot \;3}=\sqrt{\frac{3}{7}}\approx 0.654$

Taking the geometric mean of the three estimated values:


$$a_{14}\approx \sqrt[{3}]{4.24\;\cdot \;3.16\;\cdot \;0.654}\approx \sqrt[{3}]{8.762}\approx 2.062$$

The estimation of *a*_25_ involved the following three triads:

1. Triad (2, 4, 5): $\sqrt{a_{24}\;\cdot \;a_{45}}=\sqrt{6\;\cdot \;\frac{1}{3}}=\sqrt{2}\approx 1.414$
2. Triad (2, 3, 5): $\sqrt{a_{23}\;\cdot \;a_{35}}=\sqrt{4\;\cdot \;9}=\sqrt{36}=6$
3. Triad (1, 2, 5): $\sqrt{a_{12}\;\cdot \;a_{15}}=\sqrt{3\;\cdot \;\frac{1}{7}}=\sqrt{\frac{3}{7}}\approx 0.654$

Taking the geometric mean of the three estimated values:


$$a_{25}\approx \sqrt[{3}]{1.414\;\cdot \;6\;\cdot \;0.654}\approx \sqrt[{3}]{5.541}\approx 1.77$$

After estimating the missing data imputation, the updated matrix ${\text{A}}^{\left(0\right)}$ becomes:


$$A^{\left(0\right)}=[\begin{array}{ccccc} 1 & 3 & 5 & 2.062 & \frac{1}{7}\\ \frac{1}{3} & 1 & 4 & 6 & 1.77\\ \frac{1}{5} & \frac{1}{4} & 1 & 2 & \frac{1}{9}\\ \frac{1}{2.062} & \frac{1}{6} & \frac{1}{2} & 1 & \frac{1}{3}\\ 7 & \frac{1}{1.77} & 9 & 3 & 1 \end{array}]$$

The iterative algorithm is executed to minimize the global inconsistency of the matrix, progressively updating each element *a*_*ij*_ of the matrix.

The update of each element is performed according to the formula:


$$a_{ij}^{\left(t+1\right)}=a_{ij}^{\left(t\right)}+\eta \;\cdot \;\left(\frac{1}{3}\sum_{k\neq i,j}\frac{a_{ik}^{\left(t\right)}\;\cdot \;a_{kj}^{\left(t\right)}}{a_{ij}^{\left(t\right)}}-a_{ij}^{\left(t\right)}\right)$$

where:

– η is the learning rate, set to η=0.1.
– The sum is computed for all k≠i,j, representing the correction based on the triads involving *a*_*ij*_.

The pseudocode for the iterative algorithm, which refers to python cod *1#* in https://doi.org/10.5281/zenodo.15267213, is the following, while the explicit calculations of the inconsistencies obtained following this algorithm are present in *Appendix A*:

**Algorithm 1. Biomimetic PCM optimization (pseudocode).**

**Input:**

1: $𝐀\in {\mathbb{R}}^{n\times n}$: Initial PC matrix with missing/inconsistent

entries

2: η: Learning rate

3: *ε*: Convergence threshold

4: max_iter: Maximum iterations

**Output:**

5: ${𝐀}^{\prime }\in {\mathbb{R}}^{n\times n}$: Optimized PC matrix

6: **procedure** BIOMIMETICREGENERATION (𝐀,η,ϵ,max_iter)

7:   Initialize missing *a*_*ij*_ using geometric means of valid

triads:


$$a_{ij}^{\left(0\right)}={\left(\underset{k\neq i,j}{\prod }a_{ik}a_{kj}\right)}^{1/(n-2)}$$

8:   ${𝐀}_{\text{current}}\leftarrow 𝐀$

9:   t←0

10:   **while** t<max_iter **do**

11:    ${𝐀}_{\text{new}}\leftarrow \text{copy}({𝐀}_{\text{current}})$

12:    **for** *i* from 0 to *n*–1 **do**

13:     **for** *j* from *i* + 1 to *n*–1 **do**

14:      sum_term←0

15:      **for** *k* from 0 to *n*–1 **do**

16:       **if** k≠i∧k≠j **then**

17:        $\text{sum\_term}\leftarrow \text{sum\_term}+\frac{{𝐀}_{\text{current}}[i,k]\;\cdot \;{𝐀}_{\text{current}}[k,j]}{{𝐀}_{\text{current}}[i,j]}$

18:       **end if**

19:      **end for**

20:      $\Delta a_{ij}\leftarrow \eta \;\cdot \;\left(\frac{\text{sum\_term}}{n-2}-{𝐀}_{\text{current}}[i,j]\right)$

21:      Update *a*_*ij*_ and enforce reciprocity:


$${𝐀}_{\text{new}}[i,j]\leftarrow {𝐀}_{\text{current}}[i,j]+\Delta a_{ij}$$

$${𝐀}_{\text{new}}[j,i]\leftarrow \frac{1}{{𝐀}_{\text{new}}[i,j]}$$

22:     **end for**

23:    **end for**

24:    Compute difference between iterations:


$$\text{diff}\leftarrow \max_{i,j}\left|{𝐀}_{\text{new}}[i,j]-{𝐀}_{\text{current}}[i,j]\right|$$

25:    **if** diff<ϵ **then**

26:     **return** ${𝐀}_{\text{new}}$

27:    **end if**

28:    ${𝐀}_{\text{current}}\leftarrow {𝐀}_{\text{new}}$

29:    t←t+1

30:   **end while**

31:   **return** ${𝐀}_{\text{current}}$

32: **end procedure**

### 4.3 The final PC matrix and its global inconsistency

The final matrix, obtained at the end of the minimization process (discussed in *subsection A.1*, see *Appendix A*), using the python code *2#* (present in https://doi.org/10.5281/zenodo.15267213), is the following:


$$A^{\left(\text{final}\right)}=[\begin{array}{ccccc} 1 & 1.17322357 & 2.40769248 & 2.55690073 & 1.56817393\\ 0.85235246 & 1 & 2.05398016 & 2.44533255 & 1.36450547\\ 0.41533543 & 0.48685962 & 1 & 1.14086518 & 0.66451811\\ 0.39109848 & 0.40894233 & 0.87652776 & 1 & 0.58520661\\ 0.63768437 & 0.73286625 & 1.50484987 & 1.70879821 & 1 \end{array}]$$

The global inconsistency *I*(*A*) converges to the value I(A)≈0.3.

The algorithm has significantly improved the global inconsistency with respect to the matrix ${\text{A}}^{\left(9\right)}$.

In fact, the inconsistencies of the triads of the matrix ${\text{A}}^{\left(9\right)}$ are:

$K_{123}\approx 0.177$;   $K_{124}\approx 0.446$;   $K_{125}\approx 0.237$;   $K_{134}\approx 0.281$;   $K_{135}\approx 0.208$;

$K_{145}=\approx 0.01$;   $K_{234}=\approx 0.078$;   $K_{235}\approx 0.145$;   $K_{245}\approx 0.286$;   $K_{345}\approx 0.094$,

obtaining I(A)≈1.96.

## 5 Conclusions and future research

The proposed biomimetic model for PCs represents a mathematically rigorous method for computing inconsistencies and missing data (elements) PC matrices. By integrating biological principles of tissue regeneration with numerical optimization techniques, the proposed algorithm reduces inconsistencies in PC matrices, thereby enhancing the reliability of decisions made based on them.

In future research, we plan to analyze the use of orthogonalization [21] as observed in biological systems such as the orthogonal positioning of plants. We will also explore adding a rating scale to our biomimetic model presented in [35]. Furthermore, we intend to investigate the application of tensors to simplify the computation of multidimensional relationships within PC matrices. This could lead to substantial changes in the traditional approach of comparing only two elements at a time. By using tensors, we can model multiple dimensions, such as the comparisons between three or more parameters of a biomimetic system, which may include biological, mechanical, and environmental properties. The application of tensors would also simplify computations needed to reduce inconsistency at multiple levels and variables.

In general, solving complex human problems through a biomimetic approach is of considerable importance for our civilization. Our future research will focus on exploring the origins of reasoning by leveraging biomimetic principles, as suggested in our previous work [36].

## Appendix A

1. *Matrix update*: At each iteration, the update-matrix function is called to compute the new PC matrix $A_{\text{new}}$.

2. *Difference checking*: The difference between the current PC matrix and the updated PC matrix is computed using the matrix-difference function. If this difference is less than the convergence threshold ζ, the cycle stops (the matrix is considered converged).

3. *Current PC matrix updating*: If convergence is not reached, the updated PC matrix $A_{\text{new}}$ becomes the new current matrix *A*, and the iteration loop repeats.

4. *Iteration increment*: In each iteration loop, the iteration counter increases by 1. If the maximum number of iterations (max-iterations) is reached, the cycle stops regardless of the computed difference.

After nine iterations, the values of inconsistency *K*_*ii*_ drop to a very low level of inconsistency. The final result of the matrix is as follows:


$$A^{\left(9\right)}=[\begin{array}{ccccc} 1 & 1.4679 & 2.5258 & 2.2416 & 1.4707\\ 0.6812 & 1 & 2.0896 & 2.7550 & 1.3139\\ 0.3959 & 0.4786 & 1 & 1.2240 & 0.7351\\ 0.4461 & 0.3630 & 0.8170 & 1 & 0.6627\\ 0.6799 & 0.7611 & 1.3604 & 1.5090 & 1 \end{array}]$$

1. Triad (1,2,3):
  $$a_{12}=1.4679,\;a_{23}=2.0896,\;a_{13}=2.5258$$
  $$Kii=\left|1-\frac{a_{13}}{a_{12}\;\cdot \;a_{23}}\right|\approx 0.177$$
2. Triad (1,2,4):
  $$a_{12}=1.4679,\;a_{24}=2.7550,\;a_{14}=2.2416$$
  $$Kii=\left|1-\frac{a_{14}}{a_{12}\;\cdot \;a_{24}}\right|\approx 0.446$$
3. Triad (1,2,5):
  $$a_{12}=1.4679,\;a_{25}=1.3139,\;a_{15}=1.4707$$
  $$Kii=\left|1-\frac{a_{15}}{a_{12}\;\cdot \;a_{25}}\right|\approx 0.237$$
4. Triad (1,3,4):
  $$a_{13}=2.5258,\;a_{34}=1.2240,\;a_{14}=2.2416$$
  $$Kii=\left|1-\frac{a_{14}}{a_{13}\;\cdot \;a_{34}}\right|\approx 0.281$$
5. Triad (1,3,5):
  $$a_{13}=2.5258,\;a_{35}=0.7351,\;a_{15}=1.4707$$
  $$Kii=\left|1-\frac{a_{15}}{a_{13}\;\cdot \;a_{35}}\right|\approx 0.208$$
6. Triad (1,4,5):
  $$a_{14}=2.2416,\;a_{45}=0.6627,\;a_{15}=1.4707$$
  $$Kii=\left|1-\frac{a_{14}\;\cdot \;a_{45}}{a_{15}}\right|\approx 0.01$$
7. Triad (2,3,4):
  $$a_{23}=2.0896,\;a_{34}=1.2240,\;a_{24}=2.7550$$
  $$Kii=\left|1-\frac{a_{23}\;\cdot \;a_{34}}{a_{24}}\right|\approx 0.078$$
8. Triad (2,3,5):
  $$a_{23}=2.0896,\;a_{35}=0.7351,\;a_{25}=1.3139$$
  $$Kii=\left|1-\frac{a_{25}}{a_{23}\;\cdot \;a_{35}}\right|\approx 0.145$$
9. Triad (2,4,5):
  $$a_{24}=2.7550,\;a_{45}=0.6627,\;a_{25}=1.3139$$
  $$Kii=\left|1-\frac{a_{25}}{a_{24}\;\cdot \;a_{45}}\right|\approx 0.286$$
10. Triad (3,4,5):
  $$a_{34}=1.2240,\;a_{45}=0.6627,\;a_{35}=0.7351$$
  $$Kii=\left|1-\frac{a_{35}}{a_{34}\;\cdot \;a_{45}}\right|\approx 0.094$$

The iterative algorithm has significantly reduced the local inconsistency of the matrix after a limited number of iterations. The limit of inconsistency was analyzed in [22] and the number of iterations in [37].

## A.1 Minimization of *I*(*A*) using gradient descent

We proceed with minimizing *I*(*A*) using gradient descent, applying this procedure until the global inconsistency, *I*(*A*), drops to the threshold of approximately 0.3.

The update of each *a*_*ij*_ follows this rule:


$$a_{ij}^{\left(t+1\right)}=a_{ij}^{\left(t\right)}-\eta \;\cdot \;\frac{\partial I(A)}{\partial a_{ij}}$$

where $\frac{\partial I(A)}{\partial a_{ij}}$ is the derivative of *I*(*A*) with respect to *a*_*ij*_, obtained by summing the contributions of all triads involving *a*_*ij*_.

Below is the pseudocode (which refers to the Python code *2#* in https://doi.org/10.5281/zenodo.15267213), for global inconsistency minimization *I*(*A*):

**Algorithm 2. Global inconsistency minimization (pseudocode).**

**Input:** Initial PC matrix $A\in {\mathbb{R}}^{n\times n}$, learning rate η, maximum

iterations max_iter, stopping threshold ϵ

**Output:** Optimized PC matrix $A^{\prime }\in {\mathbb{R}}^{n\times n}$ with $I(A^{\prime })\leq \epsilon$

1: **Initialization:**

2: $A_{\text{current}}\leftarrow A$

3: t←0

4: **while** t<max_iter **do**

5:   $A_{\text{new}}\leftarrow \text{copy}(A_{\text{current}})$

6:   **for** *i* from 0 to *n*–1 **do**

7:    **for** *j* from *i* + 1 to *n*–1 **do**

8:     sum_term←0

9:     **for** *k* from 0 to *n*–1 **do**

10:      **if** k≠i∧k≠j **then**

11:       $\text{term}\leftarrow \frac{A_{\text{current}}[i,k]\;\cdot \;A_{\text{current}}[k,j]}{A_{\text{current}}[i,j]}$

12:       sum_term←sum_term+term

13:      **end if**

14:     **end for**

15:     Compute correction term:


$$\Delta a_{ij}\leftarrow \eta \;\cdot \;\left(\frac{\text{sum\_term}}{n-2}-A_{\text{current}}[i,j]\right)$$

16:     Update *a*_*ij*_:

17:     $A_{\text{new}}[i,j]\leftarrow A_{\text{current}}[i,j]+\Delta a_{ij}$

18:     Enforce reciprocity:

19:     $A_{\text{new}}[j,i]\leftarrow \frac{1}{A_{\text{new}}[i,j]}$

20:    **end for**

21:   **end for**

22:   Compute $I_{\text{new}}\leftarrow I(A_{\text{new}})$

23:   **if** $I_{\text{new}}<\epsilon$ **then**

24:    **return** $A_{\text{new}}$, $I_{\text{new}}$

25:   **end if**

26:   $A_{\text{current}}\leftarrow A_{\text{new}}$

27:   t←t+1

28: **end while**

29: Compute $I_{\text{final}}\leftarrow I(A_{\text{current}})$

30: **return** $A_{\text{current}}$, $I_{\text{final}}$

## Appendix B: Dimensionality reduction with PCA for large pairwise comparison matrices

This appendix demonstrates how Principal Component Analysis (PCA) can be used to reduce the size of a large pairwise comparison (PC) matrix while preserving its essential structure. The reduced matrix is then stabilized using the biomimetic algorithm described in the paper, achieving low inconsistency (*I*(*A*)) in fewer iterations.

### B.1 PCA-based reduction

The objective is reduce a n×n PC matrix to a smaller k×k matrix (*k*<*n*) while retaining key priorities.

The steps are:

- 1. Symmetrize the matrix:
  $$A_{\text{symmetric}}=\frac{A+A^{T}}{2}$$
  This ensures the matrix is symmetric, a requirement for PCA.
- 2. Apply PCA: Extract *k* principal components to capture the most variance in the data.
- 3. Cluster components: Use *k*-means clustering to group the original elements into *k* clusters.
- 4. Form Reduced Matrix: For each pair of clusters (*i*,*j*), compute the geometric mean of all pairwise comparisons between elements in clusters *i* and *j*.

### B.2 Biomimetic algorithm on reduced matrix

The reduced k×k matrix is stabilized using the algorithm from the paper:

- 1. Update rule:
  $$A_{\text{new}}[i,j]=A_{\text{current}}[i,j]+\eta \left(\frac{\sum_{k\neq i,j}\frac{A_{\text{current}}[i,k]\;\cdot \;A_{\text{current}}[k,j]}{A_{\text{current}}[i,j]}}{n-2}-A_{\text{current}}[i,j]\right)$$
  where η is the learning rate (set to 0.1), and *n* is the matrix size.
- 2. Stopping condition: The algorithm stops when the difference between consecutive matrices falls below ζ=0.03 or after max_iterations=4.

### B.3 Example with a 5×5 matrix from section *Example* of the paper

*Original Matrix:*


$$A_{5\times 5}=[\begin{array}{ccccc} 1 & 3 & 5 & 2.062 & 1/7\\ 1/3 & 1 & 4 & 6 & 1.77\\ 1/5 & 1/4 & 1 & 2 & 1/9\\ 1/2.062 & 1/6 & 1/2 & 1 & 1/3\\ 7 & 1/1.77 & 9 & 3 & 1 \end{array}]$$

Using the following pseudocode, Algorithm 3, (referring to the Python *3#* code, present in https://doi.org/10.5281/zenodo.15267213), which involves the reduction of dimensionality with PCA, the stabilization of the PC matrix through the iterative algorithm and finally the minimization of the global inconsistency *I*(*A*), we obtain:

- Reduced Matrix (3x3):
  $$\left[\begin{array}{ccc} 1.00 & 4.24 & 3.03\\ 0.24 & 1.00 & 0.49\\ 0.33 & 2.03 & 1.00 \end{array}\right]$$
  with initial *I*(*A*) (3x3): 0.31
- Stabilization and minimization Process:
  $$\left[\begin{array}{ccc} 1.00 & 3.19 & 2.28\\ 0.31 & 1.00 & 0.73\\ 0.44 & 1.38 & 1.00 \end{array}\right]$$
  with final *I*(*A*): 0.002 (after 4 iterations).

**Algorithm 3. PCA-Based dimensionality reduction for PC matrices (pseudocode).**

**Input:** Original PC matrix $A\in {\mathbb{R}}^{n\times n}$, target dimension *k*, learning rate

η, stopping threshold ϵ, maximum iterations max_iter

**Output:** Stabilized PC matrix $A^{\prime }\in {\mathbb{R}}^{k\times k}$, Final global inconsistency

$I_{\text{final}}$

1: Symmetrize the matrix: $A_{\text{sym}}\leftarrow \frac{A+A^{⊤}}{2}$

2: Compute top *k* eigenvectors of $A_{\text{sym}}$, project to get transformed

$\in {\mathbb{R}}^{n\times k}$

3: Apply K-means clustering to transformed, obtaining $\text{clusters}\in {\mathbb{N}}^{n}$

4: Initialize ${\text{indices}}_{0},{\text{indices}}_{1},\ldots ,{\text{indices}}_{k-1}$ as empty lists

5: **for** *p* from 0 to *n*–1 **do**

6:   c←clusters[p]

7:   Add *p* to ${\text{indices}}_{c}$

8: **end for**

9: Initialize $A_{\text{reduced}}$ as k×k matrix with diagonal entries 1

10: **for** *i* from 0 to *k*–1 **do**

11:   **for** *j* from *i* + 1 to *k*–1 **do**

12:    Collect elements $\left\{A[p,q]|p\in {\text{indices}}_{i},q\in {\text{indices}}_{j}\right\}$ from

original *A*

13:    **if** elements non-empty **then**

14:     $\mu_{ij}\leftarrow (\prod_{e\in \text{elements}}e{)}^{1/\text{len(elements)}}$

15:     $A_{\text{reduced}}[i,j]\leftarrow \mu_{ij}$

16:     $A_{\text{reduced}}[j,i]\leftarrow 1/\mu_{ij}$

17:    **else**

18:     $A_{\text{reduced}}[i,j]\leftarrow 1$, $A_{\text{reduced}}[j,i]\leftarrow 1$

19:    **end if**

20:   **end for**

21: **end for**

22: $A_{\text{current}}\leftarrow A_{\text{reduced}}$

23: t←0

24: **while** t<max_iter **do**

25:   $A_{\text{new}}\leftarrow \text{copy}(A_{\text{current}})$

26:   **for** *i* from 0 to *k*–1 **do**

27    **for** *j* from *i* + 1 to *k*–1 **do**

28:     $\text{sum\_term}\leftarrow \frac{1}{k-2}\overset{k-1}{\underset{m\neq i,j}{\underset{m=0}{\sum }}}\frac{A_{\text{current}}[i,m]\;\cdot \;A_{\text{current}}[m,j]}{A_{\text{current}}[i,j]}$

29:     $\Delta a_{ij}\leftarrow \eta \;\cdot \;(\text{sum\_term}-A_{\text{current}}[i,j])$

30:     $A_{\text{new}}[i,j]\leftarrow A_{\text{current}}[i,j]+\Delta a_{ij}$

31:     Enforce reciprocity: $A_{\text{new}}[j,i]\leftarrow 1/A_{\text{new}}[i,j]$

32:    **end for**

33:   **end for**

34:   Compute $I_{\text{new}}\leftarrow I(A_{\text{new}})$

35:   **if** $I_{\text{new}}<\epsilon$ **then**

36:    $A_{\text{current}}\leftarrow A_{\text{new}}$

37:    **break**

38:   **end if**

39:   $A_{\text{current}}\leftarrow A_{\text{new}}$

40:   t←t+1

41: **end while**

42: Compute $I_{\text{final}}\leftarrow I(A_{\text{current}})$

43: **return** $A_{\text{current}}$, $I_{\text{final}}$

### B.5 Discussion

PCA-based dimensionality reduction is a viable strategy for large PC matrices. The biomimetic algorithm efficiently stabilizes the reduced matrix, preserving the main priorities while reducing the computational burden, in fact after only 4 iterations the matrix reaches an inconsistency of 0.002. The Computational complexity has been reduced from *O*(5^3^) = 125 to *O*(3^3^) = 27, that is, from a cost of 1125 necessary operations (as seen in the *Section Example*) to only 108.
